# LncRNA MALAT1 Accelerates Cervical Carcinoma Proliferation by Suppressing miR-124 Expression in Cervical Tumor Cells

**DOI:** 10.1155/2021/8836078

**Published:** 2021-06-15

**Authors:** Tian Liang, Yuchen Wang, Yu Jiao, Shanshan Cong, Xinyan Jiang, Lina Dong, Guangmei Zhang, Dan Xiao

**Affiliations:** ^1^Department of Obstetrics and Gynaecology, The 1st Affiliated Hospital of Harbin Medical University, 150001 Harbin, China; ^2^Department of Psychiatry, Qiqihar Medical University, 161006 Qiqihar, China

## Abstract

Emerging studies have clarified the critical role of LncRNA MALAT1 in various pathological progressions. Here, we identified its positive relationship with cervical carcinoma proliferation. Cervical carcinoma has been considered as one of the most malignant tumors among female. Thus, our study was designed to investigate the underlying mechanism of LncRNA MALAT1 on cervical tumor cell proliferation. We observed that miR-124 was the potential target of LncRNA MALAT1 in cervical tumor cell lines (Hela, C-33A, Caski, and SiHa), the expression level of which is negatively correlated with LncRNA MALAT1 in cervical tumor cells, tissues of cervical patients, and mice. Gain- or loss-of-function analyses in cervical tumor cells have further verified the regulatory role of MALAT1 on miR-124. Additionally, the proliferation of cervical carcinoma was inhibited by miR-124 overexpression, whereas it was blocked by LV-MALAT1 transfection. *In vivo* assays, overexpression of miR-124, or knockdown of MALAT1 exhibited beneficial effects on tumor weight, size, and volume, together with elevating the survival rate, tightly related with the progression of cervical cancer. In conclusion, LncRNA MALAT1 disabled the effects of miR-124 as an inhibitory sponge, accelerating the progression of cervical carcinoma.

## 1. Introduction

Cervical carcinoma is one of the most life-threatening neoplasms among female, which is characterized as low survival rate at the advanced stage [1]. A recent report from the World Health Organization released that the patients with new-onset cervical carcinoma increased to over 560 thousand, and the morbidity and mortality of cervical carcinoma are 13 and 7 per 100 thousand people, respectively [2]. Accordingly, the prevalence of cervical carcinoma tends to be younger, which emphasizes the significance on the study of disease treatment [3].

Canonical treatments include surgery and chemotherapy, or their combination. With the development of precision therapy, research studies on potential molecular targets were demonstrated to play an important role in malignant diseases. Recently, LncRNAs emerge to be the focus, with high effective and multiple regulatory effects on various physiological and pathological progressions. LncRNA XLOC_009167 has been reported to serve as a diagnostic biomarker among lung-cancer patients [4]. LncRNA CDKN2BAS has been reported to promote hepatocellular carcinoma metastasis via miR-153-5p/ARHGAP18 pathways [5]; LncRNA SNHG8 was observed to regulate the process of acute myocardial infarction [6]. Also, the key role of LncRNAs on cervical carcinoma has been addressed [7–10], including PTCSC3, NEAT1, UCA1, and MALAT1. Among these LncRNAs, MALAT1 has been commonly considered as a promising therapeutical target due to its well-conserved property between species [11, 12]. MALAT1 has been found to inhibit the apoptosis and autophagy of hepatocellular carcinoma cell by targeting the microRNA-146a/PI3K/Akt/mTOR axis [13]. Besides, the relationship between MALAT1 and gynecological oncology has been gradually investigated. Researchers have clarified the regulatory effects of MALAT1 on breast cancer via inactivating prometastatic transcription factor TEAD [14], as well as promoting epithelial ovarian cancer by the PI3K-AKT pathway [15]. However, studies on cervical carcinoma are required to be further improved, and the underlying mechanism remains unclear.

LncRNAs are a series of non-protein-coding RNAs, which contain over 200 nucleotides [16]. LncRNAs function as a sponge to distinguish small regulatory RNAs such as miRNAs and subsequently regulate gene expression [17, 18]. Conservation is the most important property for LncRNAs to perform its function. Thus, in our present study, we aimed to investigate the underlying mechanism of MALAT1 in the progression of cervical carcinoma, which is characterized as good conservation. Here, we hypothesized that MALAT1 is the key mediator for the proliferation of cervical tumor cells by its potential target, miR-124, increasing the tumor growth, as well to accelerate the development during NSCLC.

## 2. Materials and Methods

### 2.1. Human Subjects and Informed Consent

Cervical carcinoma patients that were newly diagnosed received treatment at the first affiliated hospital of Harbin Medical University. The study was approved by the Institutional Ethical Board, and tumor samples were collected upon a written informed consent. The patients' information of cervical cancer is shown in Table 1. Tumor samples were extracted and analyzed for the determination of MALAT1 and miR-124 levels.

### 2.2. Animals

The protocols of animal experiment were in agreement with the Ethics Committees of Harbin Medical University. Experimental principles were fulfilled with the Guide for the Use and Care of Laboratory Animals published by the US National Institutes of Health (NIH Publication No. 85–23, revised 1996). Six-week-old BALB/c nude female mice were provided by the Animal Center of the 2nd Affiliated Hospital of Harbin Medical University (Harbin, China) and housed in a dedicated room with 12 h dark/light cycle, controlled temperature (22 ± 1°C), and constant humidity (55 ± 5%). After 1-week acclimatization, thirty nude mice bearing U14 cervical tumor xenografts were divided into five groups randomly: negative controls (LV-NC), shMALAT1, LV-MALAT1, miR-124, and miR-124 + LV-MALAT1.

### 2.3. Cell Culture and Treatment

End/E6E, Hela, Caski, C-33A, and SiHa cell lines were obtained from Shanghai Institutes for Biological Sciences (SIBS, China) and cultured in an RPMI-1640 medium (Thermo Fisher Scientific, Waltham, MA, USA) supplied with 10% fetal bovine serum (FBS, Gibco, USA) and 1% penicillin/streptomycin (100 *μ*g/ml) in 5% CO_2_ at 37°C. Before treatment, cells were incubated with the FBS-free restriction/treatment medium overnight. We followed the methods of Zhang et al. [19].

### 2.4. Cell Transfection

End/E6E, Hela, Caski, C-33A, and SiHa cells were seeded into a 6-well plate at a density of 2 × 10^6^ cells/well. At the confluence of 80%, cells were starved in a serum-free medium for 24 h before transfection. shMALAT1, MALAT1, and negative control (NC) were subcloned into pcDNA3.1 GenePharma (Shanghai, China) and then transfected with pcDNA3.1 plasmid using the Lipofect2000 transfection reagent according to manufacturer's instructions. miR-124 mimics, anti-miR-124 oligonucleotide (AMO-124), and their negative controls were transfected with the X-treme GENE siRNA Transfection Reagent (Cat. No. 04476115001, Roche, Basel, Switzerland). The medium was replaced by a regular growth medium after 6 h transfection. We followed the methods of Zhang et al. [19].

### 2.5. Real-Time PCR

Total RNA was harvested from cells using the TRIzol reagent (Invitrogen, Carlsbad, CA, USA) according to previous studies. cDNA synthesis was performed using a High-Capacity cDNA Reverse Transcription Kit (Applied Biosystems, Carlsbad, CA, USA; Cat. No. 4368814) according to the manufacturer's instructions. The levels of MALAT1 and miR-124 were determined by the SYBR Green I incorporation method and ABI 7500 fast Real-Time PCR system (Applied Biosystems, USA). GAPDH was used as internal control [19].

### 2.6. Proliferation Assay

End/E6E, Hela, Caski, C-33A, and SiHa cells were seeded in a 96-well plate. At the indicated time point, the CCK-8 reagent was added to each well (10 *μ*l/well) and continually incubated for 4 h. The optical density (OD) value (450 nm) was determined by an enzyme-linked immunosorbent assay plate reader (Bioreader) for CCK-8 assay.

In trypan blue staining, cells were resuspended and mixed with 0.1% trypan blue at room temperature for 5 min. Cell suspension was counted on an automated cell counter (Countless II FL, Invitrogen) and analyzed for the ratio of living cells [19].

### 2.7. Statistical Analysis

All values were presented as mean ± S. E. M. Statistical comparisons were performed by Student's *t*-test between two groups or one-way ANOVA for multiple comparisons. *p* < 0.05 was considered to indicate a significant difference. Data were analyzed using the GraphPad Prism 8.0 software. Correlations between MALAT1 and miR-124 were assessed by using Pearson, Spearman, and Kendall's rank correlation coefficient analyses [20].

## 3. Result

### 3.1. MALAT1 and miR-124 Expression Level Are Negatively Related in Cervical Carcinoma Patients

**Figure 1:**
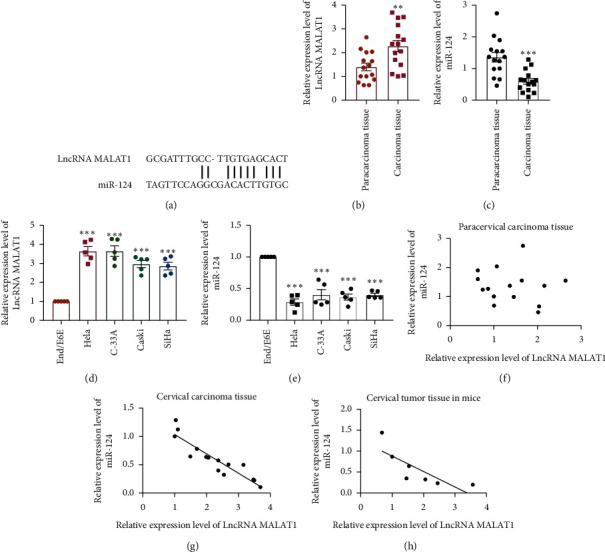
MALAT1 is negatively related to miR-124 expression in cervical tumor tissues and cell lines. (a) Complementary pairing sequence of LncRNA MALAT1 and miR-124 was evaluated by RNA22 v2 microRNA target detection (https://cm.jefferson.edu/rna22/Interactive). (b, c) LncRNA MALAT1 and miR-124 levels were detected by real-time PCR in paracarcinoma tissues and carcinoma tissues of 15 cervical tumor patients, *n* = 15, ^*∗∗*^*p* < 0.01 and ^*∗∗∗*^*p* < 0.001. (d, e) LncRNA MALAT1 and miR-124 levels were detected by real-time PCR in End1/E6E, Hela, C-33A, Caski, and SiHa cell lines, *n* = 5 in each batches, ^*∗∗∗*^*p* < 0.001 compared with the End1/E6E group. (f–h) Linear analysis was performed to detect the relationship between LncRNA MALAT1 and miR-124 levels in tissues from 15 cervical cancer patients and 7 tumor-bearing nude mice.

According to the sequence examined (Figure 1(a)) and previous studies, we hypothesized that miR-124 was the potential target of MALAT1 in regulating cervical tumor progression. To verify our hypothesis and determine the regulatory role of LncRNA MALAT1 in cervical carcinoma, we first compared the expression levels between paracarcinoma tissues and carcinoma tissues in 15 cervical cancer patients, as well as in the normal cervical epithelial cell line (End1/E6E) and cervical tumor cell lines (Hela, C-33A, Caski, and SiHa). The result showed that LncRNA MALAT1 was highly expressed in carcinoma tissues and cervical tumor cells (Figures 1(b) and 1(d)). We then further explored its potential downstream target, miR-124. The expression levels of miR-124 were downregulated in carcinoma tissues and cervical tumor cells compared with paracarcinoma tissues and End1/E6E cells (Figures 1(c) and 1(e)). These results suggested a negative relationship between LncRNA MALAT1 and miR-124. Thus, we subsequently examined LncRNA MALAT1 and miR-124 by real-time PCR in cervical carcinoma tissue compared with its paracarcinoma tissue, as well as in mice tumor tissues. The results showed that the expression levels of LncRNA MALAT1 and miR-124 were negatively related in the progression of cervical carcinoma, nor in normal conditions (Figures 1(f)–1(h) and Table 2). Thus, we concluded that the LncRNA MALAT1 expression level is negatively related to miR-124, implying their regulatory relationship in the progression of cervical carcinoma.

Three canonical correlation analyses (Pearson, Spearman, and Kendall) were utilized to illustrate the relationship between MALAT1 and miR-124.

### 3.2. MiR-124 Was Inhibited by MALAT1 in Cervical Carcinoma Cells

**Figure 2:**
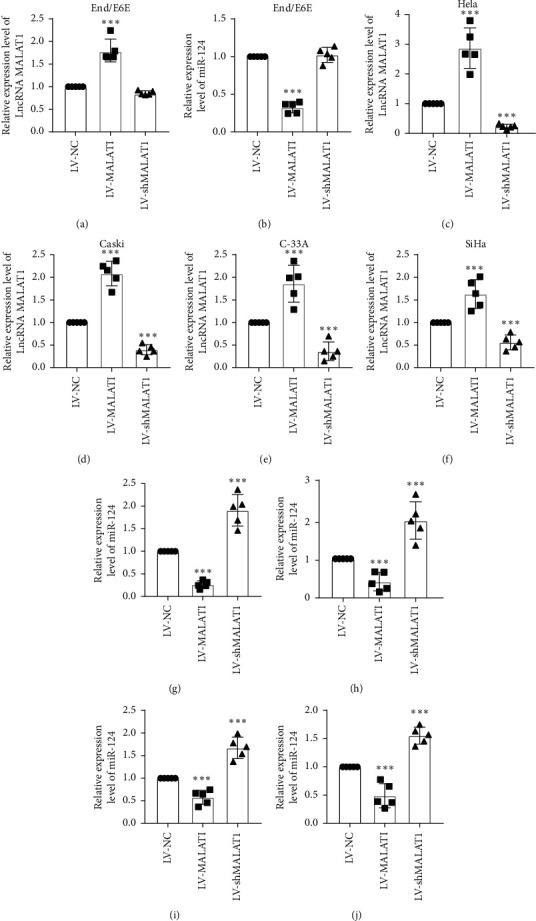
LncRNA MALAT1 regulates miR-124 in cervical carcinoma cells. (a, c–f) Transfection effectives of LV-NC (LV-negative control), LV-MALAT, and LV-shMALAT1 were determined by real-time PCR analysis, *n* = 5, ^*∗∗∗*^*p* < 0.001 compared with the LV-NC group. (b, g–j) The expression levels of miR-124 were determined by real-time PCR, *n* = 5, ^*∗∗∗*^*p* < 0.001 compared with the LV-NC group.

LncRNAs are recognized as a transcriptional regulator in various physiological and pathological processes. Generally, its inhibitory effect on miRNAs has been fully reviewed previously. To further define the relationship between LncRNA MALAT1 and miR-124, we transfected LV-MALAT1, LV-shMALAT1, and LV-NC into End/E6E, Hela, C-33A, Caski, and SiHa cells and then observed the alternation of miR-124. Results of transfection efficiency were assessed as in Figures 2(a) and 2(c)–2(f), illustrating that transfection of LV-shMALAT1 in cervical cancer cells significantly decreased the LncRNA MALAT1 level, whereas LV-MALAT1 overexpressed it compared with the End/E6E cell line. Knockdown of LncRNA MALAT1 increased the miR-124 level in cervical carcinoma cells (Figures 2(b) and 2(g)–2(j)). In contrast, overexpression of LncRNA MALAT1 inhibited the expression of miR-124. Taking together the gain- and loss-of-function results, we concluded that miR-124 was the downstream of LncRNA MALAT1 in cervical carcinoma cells.

### 3.3. LncRNA MALAT1 Promotes the Proliferation of Cervical Tumor Cells via Inhibition of miR-124

**Figure 3:**
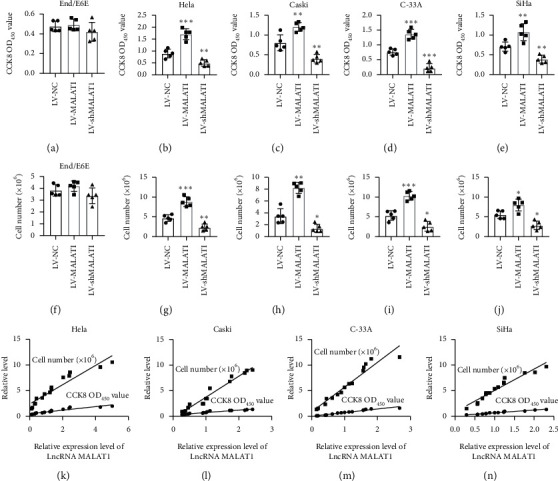
LncRNA MALAT1 elevates the proliferation of cervical tumor cells. (a, c–f) Cell viabilities of LV-NC, LV-shMALAT, and LV-MALAT1 groups were determined by CCK-8 assays, *n* = 5, ^*∗∗*^*p* < 0.01 and ^*∗∗∗*^*p* < 0.001 compared with the LV-NC group. (b, g–j) Cell numbers of LV-NC, LV-shMALAT, and LV-MALAT1 groups were determined by trypan blue staining assays, *n* = 5, ^*∗*^*p* < 0.05, ^*∗∗*^*p* < 0.01, and ^*∗∗∗*^*p* < 0.001 compared with the LV-NC group.

**Figure 4:**
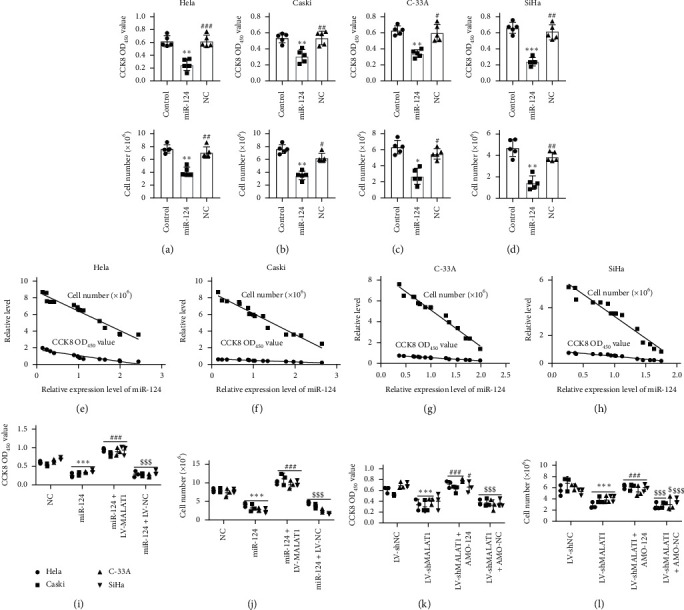
LncRNA MALAT1/miR-124 axis regulates the proliferation of cervical tumor cells. (a–d) Cell viabilities and numbers of control, miR-124, and NC (negative control) groups were determined by CCK-8 assays and trypan blue staining, *n* = 5,^*∗∗*^*p* < 0.01 and ^*∗∗∗*^*p* < 0.001 compared with the control group. ^##^*p* < 0.05, ^#^*p* < 0.01, and ^###^*p* < 0.001 compared with the miR-124 group. (e–h) Linear correlation of cervical cancer cell proliferation was analyzed with miR-124 level in Hela, Caski, C-33A, and SiHa cell lines. (i, j) Cell viabilities and numbers of NC (negative control of miR-124), miR-124, miR-124 + LV-MALAT1, and miR-124 + LV-NC groups were determined by CCK-8 and trypan blue staining assays, respectively. *n* = 3 in each batches, ^*∗∗∗*^*p* < 0.001 compared with the NC group, ^###^*p* < 0.001 compared with the miR-124 group, and ^$$$^*p* < 0.001 compared with the miR-124 + LV-MALAT1 group. (k, l) Cell viabilities and numbers of LV-shNC, LV-shMALAT1, LV-shMALAT1 + AMO-124, and LV-shMALAT1 + AMO-NC groups were determined by CCK-8 and trypan blue staining assays, respectively. *n* = 3 in each batches, ^*∗*^*p* < 0.05 and ^*∗∗∗*^*p* < 0.001 compared with the LV-shNC group, ^#^*p* < 0.05 and ^###^*p* < 0.001 compared with the LV-shMALAT1, and ^$^*p* < 0.05 and ^$$$^*p* < 0.001 compared with the LV-shMALAT1 + AMO-124 group.

In order to delineate the function of LncRNA MALAT1 in cervical carcinoma, we further gain insight into the effect on cervical tumor cell proliferation, which is the key activity during tumor growth. The results of CCK-8 and trypan blue staining assays showed that compared with noncervical cancer cell End/E6E, knockdown of LncRNA MALAT1 significantly reduced the viability and cell number of Hela, C33A, Caski, and SiHa cells, whereas overexpression of LncRNA MALAT1 increased these features (Figures 3(a)–3(j)). Linear correlation analyses showed a positive correlation between LncRNA MALAT1 and cervical cancer cell proliferation viability (Figures 3(k)–3(n)). We then examined the effects of miR-124 on the proliferation of cervical tumor cells. The results showed that miR-124 functioned against cervical cell proliferation compared with its negative control group (Figures 4(a)–4(d)). For a further proof, we performed linear correlation analyses. As expected, the levels of miR-124 are negatively related with proliferation viability of Hela, Caski, C-33A, and SiHa cervical cells (Figures 4(e)–4(h)).

**Figure 5:**
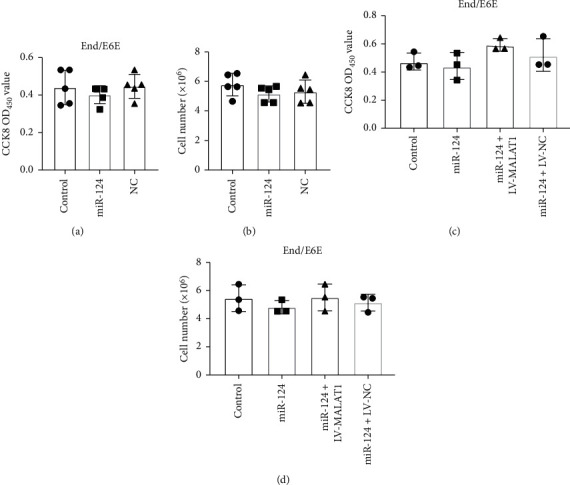
MALAT1/miR-124 axis failed to regulate End/E6E cells. (a) Cell viabilities of control, miR-124, and NC groups were determined by CCK-8 assays, *n* = 5. (b) Cell numbers of control, miR-124, and NC groups were determined by trypan blue staining assays, *n* = 5. (c, d) Cell viabilities and numbers of control, miR-124, miR-124 + LV-MALAT1, and miR-124 + LV-NC groups were determined by CCK-8 and trypan blue staining assays, respectively, *n* = 3 in each batches.

Furthermore, to illustrate the regulatory effect of the LncRNA MALAT1/miR-124 axis on cervical cell proliferation, we cotransfected miR-124 with LncRNA MALAT1. Overexpression of LncRNA MALAT1 remarkably neutralized the inhibitory effect of miR-124 on cell proliferation (Figures 4(i) and 4(j)). In addition, the inhibitory effect on cervical cell proliferation viability by knockdown LncRNA MALAT1 was also blocked by anti-miR-124 oligonucleotide (AMO-124) (Figures 4(k) and 4(l)). Additionally, we observed that the LncRNA MALAT1/miR-124 axis failed to regulate the proliferation in End/E6E cells (Figure 5) confirming the regulatory axis of LncRNA MALAT1/miR-124 on cervical tumor cell proliferation.

### 3.4. LncRNA MALAT1 Accelerates Cervical Tumor Growth by Downregulating the Expression of miR-124

**Figure 6:**
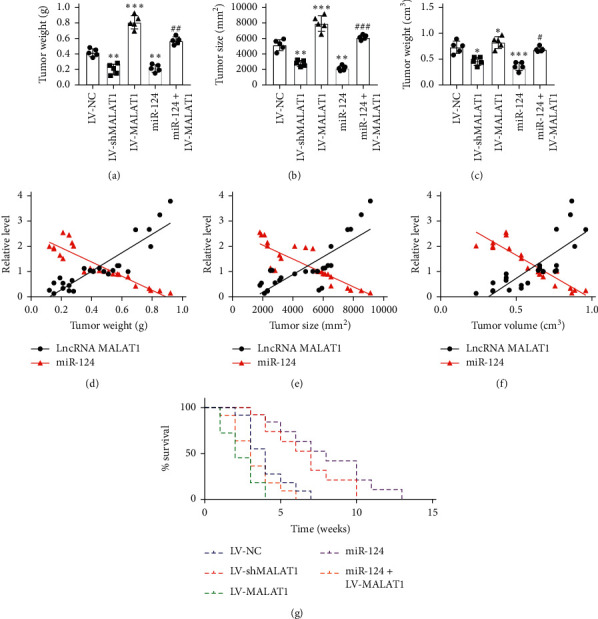
LncRNA MALAT1 accelerates tumor growth in the progression of cervical carcinoma by downregulating the expression of miR-124. (a–c) Tumor weight, size, and volume were measured in each group, *n* = 5, ^*∗*^*p* < 0.05, ^*∗∗*^*p* < 0.01, and ^*∗∗∗*^*p* < 0.001 compared with the LV-NC group. ^#^*p* < 0.05, ^##^*p* < 0.01, and ^###^*p* < 0.001 compared with the miR-124 group. (d–f) Linear correlation analysis between tumor weight, size, and volume versus LncRNA MALAT1 or miR-124. (g) Survival percentage was calculated throughout 8-week feeding.

To further underpin the effect of the LncRNA MALAT1/miR-124 axis on cervical tumor growth, we used tumor-bearing mice with tumor tissue index and survival rate. U14 cervical cancer cells were transfected with negative control vector, LV-shMALAT1, LV-MALAT1, miR-124, and miR-124 + LV-MALAT1. Mice were sacrificed for tumor tissue extraction following 8 weeks of feeding. Tumor index including tumor weight, size, and volume were calculated as in Figures 6(a)–6(c). Linear correlation analyses were also performed with tumor index and the levels of LncRNA MALAT1, as well as miR-124 (Figures 6(d)–6(f)). LncRNA MALAT1 functioned as a tumor growth regulator increased tumor growth, while miR-124 acted as a negative catalyst, suppressing tumor weight, size, and volume. In addition, overexpression of LncRNA MALAT1 abolished the effect of miR-124, suggesting its underlying mechanism of tumor growth promotion. Also, mice with LncRNA-MALAT1-overexpressed tumor tissues showed the lowest survival rate compared, different from the miR-124-transfected group, which showed the beneficial effects on mice survival. LncRNA MALAT1 overexpression blocked the elevation of survival rate in the miR-124-transfected group, aggravating cervical carcinoma (Figure 6(g)). These results suggested that LncRNA MALAT1 in cervical tumor tissues accelerated the development of tumor growth by reducing miR-124 expression, leading a relatively low survival numbers via promoting tumor cell proliferation. Taken together, LncRNA MALAT1/miR-124 is a key axis in cervical carcinoma, exerting regulatory effects on the proliferation of the cervical tumor cell.

## 4. Discussion

Cervical carcinoma defines as a malignancy which develops in the epithelium of the cervix and is responsible for 10–15 % of all female cancer-related deaths, carrying high risks of morbidity and mortality. Worldwide, there are about 500,000 new cases of cervical cancer each year, accounting for 5% of all new cases of cancer. A large number of studies in recent years have shown that the onset age of cervical cancer is becoming younger, suggesting a great need and urgency in searching novel treatment targets and prognosis biomarkers to improve the survival rate of cervical cancer patients [21]. However, the regulatory factor in the progression of cervical carcinoma remains uncomprehensive. Thus, research related to cervical carcinoma requires further investigation.

Many LncRNAs are gradually recognized as key regulators in various biological processes and took vitally an effect on the oncogenesis [22] and progression of cervical cancer [28]. Increased expression of LncRNA HOTAIR has been observed in cervical cancer tissues [23] and correlated with the FIGO stage, lymphatic metastasis, and size of tumor, as well as invasive depth, indicating its involvement in cervical cancer progression [24]. Additionally, LncRNA HOTAIR is positively related to tumor recurrence and epithelial to mesenchymal transition (EMT) [25]. LncRNA GAS5 was downregulated in cervical cancer tissues, significantly correlated to advanced cancer progression, and identified as a separate biomarker for forecasting the clinical states of patients in cervical cancer [26]; LncRNA TUSC8 was dramatically reduced in cervical cancer and linked to the FIGO stage, size of tumor, and squamous cell carcinoma antigen [27]; LncRNA LET was also observed to be downregulated in cervical tumor. Knockdown of LncRNA LET contributed to worse overall survival [28]. Research studies on LncRNAs suggest their potential to act as a prognosis marker and treatment target of cervical cancer.

Among these LncRNAs, metastasis-associated lung adenocarcinoma transcript 1 (MALAT1) was commonly considered as a target for antimetastatic therapy in cancer, which is associated with solid tumor progression [29, 30]. Previous studies have uncovered its prognostic property for the metastasis in lung cancer [31, 32]. Furthermore, MALAT1 promoted EMT via increasing Snail in cervical tumor cells [33], implying the aggregating effect of MALAT1 on cervical carcinoma.

The underlying mechanisms of LncRNAs have been broadly reviewed [34–36]. Mainly, LncRNAs may function as competing RNA in managing the activities and biological functions of miRNAs, which acted as miRNA sponges [36, 37]. Recently, it was reported that lncRNA-MEG3 served as a cancer suppressor via lessening the expression of miR-21 in cervical cancer [38]; MALAT1 increased cell colony formation and cell cycle regulation and suppressed cell apoptosis through sponging miR-145 in cervical cancer [39]. These findings could furnish certain evidence on the lncRNA-miRNA interaction in carcinogenesis of cervical cancer. Proliferation of cervical tumor cells is the main process of carcinoma formation and progression. However, the mechanism of MALAT1 in accelerating the proliferation remains unclear.

In the current study, we highlighted three observations. First, we revealed the negative correlation between MALAT1 and miR-124 in cervical tumor tissues and cell lines, verifying their regulatory relationship. Second, a significant alternation of MALAT1/miR-124 axis was found in the proliferation of cervical tumor cells, suggesting the regulatory role of the MALAT1/miR-124 axis on cell proliferation. Third, the MALAT1/miR-124 axis regulates cervical cell proliferation, at least in part, associated with the development of tumor growth and survival rates.

Additionally, our research proposed the consideration whether specifically inactivating MALAT1 expression in cervical tumor via gene engineering tools could ameliorate mortality and morbidity of cervical carcinoma, or other cancers, providing more beneficial effects to improve the outcomes.

## 5. Conclusions

In this study, we found that LncRNA MALAT1 accelerates tumor growth during cervical carcinoma via inhibiting miR-124. Our findings suggest that MALAT1 could be recognized as a potential target to improve clinical effects in the progression of cervical carcinoma.

## Figures and Tables

**Table 1:** Sample information of 15 cervical cancer patients.

Clinical parameters	*n* (%)
Age	
<50	4 (26.67)
≥50	11 (73.34)

Histologic type	
SCC	13 (86.67)
ADC	2 (13.33)

Histologic grade	
Well-intermediate differentiation	10 (66.67)
Poor differentiation	5 (33.33)

LNM	
No	12 (80)
Yes	3 (20)

SCC: squamous cell carcinoma; ADC: adenocarcinoma; LNM: lymph node metastasis.

**Table 2:** The correlation analyses of LncRNA MALAT1 and miR-124.

Analyses	*R* value	*p*
Pearson	−0.968	<0.01
Spearman	−0.974	<0.01
Kendall	−0.959	<0.01

## Data Availability

The research data used to support the findings of this study are available from the corresponding author upon request.
